# Prediction of Offensive Possession Ends in Elite Basketball Teams

**DOI:** 10.3390/ijerph18031083

**Published:** 2021-01-26

**Authors:** Kęstutis Matulaitis, Tomas Bietkis

**Affiliations:** Department of Coaching Science, Lithuanian Sports University, Sporto 6, 44221 Kaunas, Lithuania; tombie@stud.lsu.lt

**Keywords:** coaching, performance analysis, end of the ball possession, predictive model

## Abstract

In basketball, the end of the ball possession has been described as one of the most important determinants of successful offensive play by a team. The present study aimed to: (i) investigate outcomes according to the play types of ends of the ball possession; (ii) find the most efficient ball possessions during the game; (iii) predict most efficient ends of the ball possession by time in an elite basketball competition. The sample was composed of 38,640 situations of ends of the ball possession from 240 games of the 2017–2018 regular season of the men’s Euroleague that were quantitatively analyzed. According to the results, the predictive model can be used in modern basketball. The most efficient ends of the ball possession are the 2-point field goals on the fast break (78.2%), cuts (64.8%), pick and roll (P&R) screener (61.5%), and transition and offensive rebound (57.4%) situations. This information allows a better collective understanding of basketball, and it could be a great tool to use for coaches to prove which tactical solutions are to be considered when improving offense and defense strategies. It also contributes to the design of precise practice tasks of the coach that improve the game.

## 1. Introduction

Nowadays coaches and basketball scientists search for the best way to predict the offensive plays of the opponent’s team for the upcoming game [1,2]. However, understanding of tactical elements through collective behavior assessment is essential to improve performance, supporting the training process, and preparation for the match [3]. It allows to detect dynamics of the game and quantify its effectiveness, and players’ performance evaluation becomes one of the main aims for coaches [1,4,5]. The more players move and cooperate, the more likely the attack succeeds. Players dynamics are determined by specific offensive aims such as creating free space to pass and shoot, enhancing effective scoring options, and minimizing defensive pressure [6].

Through the categorization of player interactions in matches, research studies have indentified actions used in the offense: handoff [7], post up [7], spot up [7], pick and roll ball handler [8,9,10], pick and roll screener [8,9,10], isolation [7,10,11], cuts [7], offensive rebound [12], offscreen [7,8,13,14], transition [10,15], fast break [16], and other actions [7,17]. These techniques have been implemented to analyze and model offensive and defensive interactions of elite teams [14,18,19].

The prediction of set plays is important to the coach’s game preparation [7]. Set play is strategically planned to create best opportunities in best areas to get open shots and to score points [20]. The coach’s prediction of the opponent team’s offensive strategies can be crucial to the result of the game [7]. Understanding how teams generate successful scoring opportunities is practically and theoretically pivotal [21]. According to the Zukolo et al. [7] study, there is a lack of research that proves that it is crucial to gain a full insight into the types of finishing actions and modalities that make the team successful. Results of this research will help to find ways to improve their training and coaching processes and the quality of the game. Therefore, identifying offensive trends and game patterns are vital to the preparation of training sessions aimed at improving players’ tactical performance and decision-making according to specific game situations and constraints [10,22].

Significant studies of performance analysis in basketball are trying to find which factors are the key determinants on the outcome of the game [19]. Therefore, when improving offensive strategies or when analyzing the ends of possessions to improve the overall game of the team, coaches should aim to improve the game-reading ability of the players [2], decision-making ability [23], and anticipation ability [24,25].

This study aims to: (i) investigate outcomes according to the play types of ends of the ball possession; (ii) find the most efficient ball possessions during the game; (iii) predict the most efficient ends of the ball possession by time in an elite basketball competition.

## 2. Materials and Methods

### 2.1. Sample and Data Collection

Game data of the ends of the ball possession from the 2017–2018 Euroleague (teams *n* = 16; games *n* = 240) season were obtained from a publicly-accessed Euroleague website (http://tv.euroleague.net/). All ends of possession of the Euroleague’s regular-season games *n* = 38,640, efficient *n* = 17,742 (45.9% of the total), and inefficient, *n* = 20,898 (54.1% of the total) were analyzed. Every single end of the ball possession was arranged based on the following outcomes: 2-point field goals (both efficient and inefficient), 3-point field goals (both efficient and inefficient), received fouls (efficient), and turnovers (inefficient).

The current study protocol was approved and followed by the guidelines stated by the Ethics Committee of the of Lithuanian Sports University (Ethical code number BE-2–55, and date of approval 27 December 2011), based in Kaunas (Lithuania) and conformed to the recommendation of the Declaration of Helsinki adopted by the Word Medical Association [26].

### 2.2. Situational Variables

The variables examined included independent variables (Table 1). Definitions of these variables can be found in the previous studies [7,15,27,28]. Each variable has its values that allow it to accurately define the actions and situations analyzed (Table 1). The data of the ends of the ball possession were analyzed under the following, based on the outcomes and based on the type of the end of the ball possession.

The effectiveness of ball possession was transformed into a dichotomous dependent variable: the successful ball possessions (when the offensive team scored a 2- or a 3-point field goals, or received a foul), and the unsuccessful ball possessions (when the offensive team missed a 2- or 3-point field goals, blocked a shot, committed a foul, made a turnover, or made any other rule violation) [17].

### 2.3. Reliability

We collected data from 240 games and they were analyzed through systematic observation by two experienced analysts (basketball coaches with more than five years of experience in basketball performance analysis). The reliability of the data was assessed regarding to the actual agreement and Cohen’s kappa [29]. Intra-rater test-retest reliability was examined after 10 days by assessing 16 variables randomly selected from 5 games (about 80.5 end of the ball possession per game). The obtained results showed very good kappa values (range = 0.92–0.95) for intra-observer reliability, while inter-observer reliability showed very good values (range = 0.89–0.97) according to Altman (1991) [30].

### 2.4. Statistical Analysis

A total of 38,640 samples of distribution of the ends of the ball possession from 240 games were analyzed. The duration of every possession was calculated according to the shot clock. The descriptive analysis was performed using means of a count of the event and standard deviation. Also, the confidence interval (CI) is used, which shows the range (lower and upper) in which, with a slight probability, the real indicators exist.

In order to compare the differences between effective and ineffective types of ends of the ball possession, nonparametric Pearson’s Chi-squared and Mann–Whitney U tests were applied, and the statistical significance level was set at *p* < 0.05. The effect sizes (ESs) were calculated using Cramer’s *V* test, and their interpretation was based on the following criteria: 0.10 = small effect, 0.30 = medium effect, and 0.50 = large effect [29].

We used Chi-square Automatic Interaction Detector (CHAID) decision tree model method to try and create a predictive Euroleague’s efficient end of the ball possession model that shows a multilevel interaction among factors. The model consists of 27 nodes, 19 of them are terminal nodes, and depth of the model—3 levels. Insignificant variables were deleted from the final model, but all inspected variables were included in the final model. The CHAID decision tree model distinguishes the most important attributes out of many independent attributes and allows us to find out how independent attributes (Outcomes, End of the ball possession, Time, (s)) affect dependent attributes (efficient end of possession). The model was validated using the Split-sample validation method and classified forming parent nodes from no less than 500 cases (Minimum Cases in Parent Node in case) and subsidiary nodes out of no less than 250 cases (Minimum Cases in Child Node in case). Such a method allows us to efficiently analyze a large number of denominative factors that are difficult to analyze using only associative analysis (Chi-Square test). Classification trees model non-linear phenomena, and also provide visual data easily interpreted by non-analysts [31,32,33]. All statistical tests were performed using the software package IBM SPSS version 23.0 for Windows (IBM Corp., Armonk, NY, USA).

## 3. Results

### 3.1. Difference between Efficient and Inefficient Ends of Possession

Comparisons in variables among different ends of the ball possession from four outcomes according to twelve ends of the ball possessions are presented in Table 2. During all Euroleague regular-season games, the players end of the ball possessions were most often and most efficiently from 2-point field goals, plays (efficient 52.9%; inefficient 47.1%). For the 3-point field goals, there were successful 37.3% and unsuccessful 62.7% ends of ball possession. Various ends of possession were stopped: fouls received 3891 times, and 5333 ended in turnovers. After the Chi-Square test, it was determined that the following ends of possession (2-point field goals, 3-point field goals, fouls received, and turnover) and successful completion are related not by chance $\left(\chi^{2}=9796.744;p<0.001\right)$, and the effect size of these two attributes are very strong Cramer’s V (ES = 0.504). It was also determined that different ends off ball possession that ended in a shot are related $\left(\chi^{2}=1591.193;p<0.001\right)$, and the effect size of these two attributes is of medium strength Cramer’s V (ES = 0.203).

The results show that significant differences were determined using the Mann–Whitney U test by analyzing the differences and by comparing all ends of possession based on all outcomes in general, both efficient and inefficient. It was found that some ends of possession are way more efficient and the mean rank of those ends of possession was higher than of those that ended inefficiently (*p* < 0.001): fast break (mean rank = 327.28, successful; mean rank = 153.72, unsuccessful; ES = 0.64 large), cut (mean rank = 320.80, successful; mean rank = 160.20, unsuccessful; ES = 0.58 large), P&R screener (mean rank = 286.40, successful; mean rank = 194.60, unsuccessful; ES = 0.33 medium), and offensive rebound (mean rank = 286.40, successful; mean rank = 194.60, unsuccessful; ES = 0.28 small).

Significantly different results were determined using the Mann–Whitney U test by comparing the distribution of different ends of possession that ended in a 2-point field goal (*p* < 0.001). So the outcomes of different ends of possession in a 2-point field goal matched the mean rank and it was higher than of inefficient ends of ball possession in a 2- point field goals: fast break (mean rank = 321.28, successful; mean rank = 159.72, unsuccessful; ES = 0.60 large), cuts (mean rank = 313.39, successful; mean rank = 167.61, unsuccessful; ES = 0.53 large), P&R screener (mean rank = 293.97 successful; mean rank = 187.03, unsuccessful; ES = 0.39 medium), transition (mean rank = 273.53, successful; mean rank = 207.48, unsuccessful; ES = 0.24 small), and offensive rebound (mean rank = 271.63, successful; mean rank = 209.37, unsuccessful; ES = 0.23 small). However, ends of the ball possession from spot-up, P&R ball handler, isolation, and off-screen situations more often ended up inefficiently rather that efficiently.

Another important result is that ends of the ball possession that ended in the 3-point field goal area much more often ended in shots missed rather than shots made. Its mean rank was higher than of efficient ends of ball possession (*p* < 0.001). The ends of the ball possession that are most often chosen by the players to finish actions during team-tactical plays should be noted: spot-up (mean rank = 164.32, successful; mean rank = 316.68, unsuccessful; ES = 0.55 large), isolation (mean rank = 175.08, successful; mean rank = 305.92, unsuccessful; ES = 0.49 medium), and P&R ball handler (mean rank = 181.68, successful; mean rank = 299.33, unsuccessful; ES = 0.43 medium). Therefore, regardless of the end of the ball possession, possessions in 3-point field goals more often ended inefficiently rather than efficiently.

### 3.2. Predictive Model of Successful Ends of the Ball Possession

**Figure 1:**
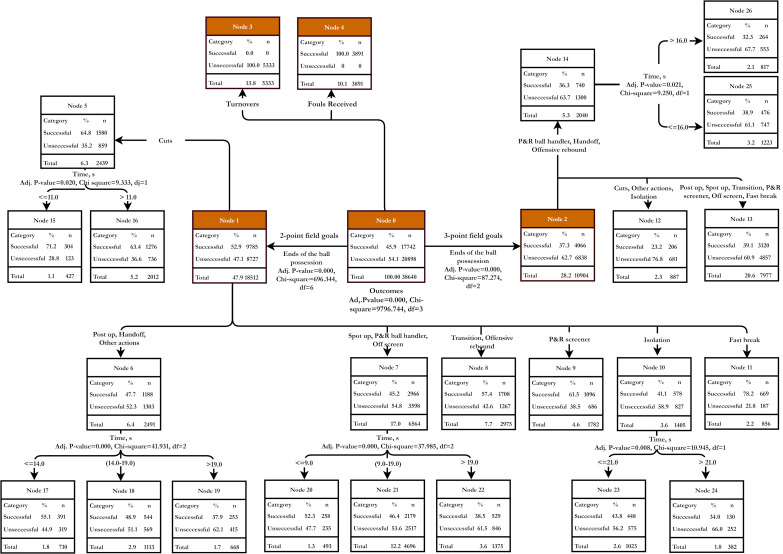
Chi-square Automatic Interaction Detector (CHAID) tree describing frequency effectiveness (%) of the predictive model of successful ends of the ball possession.

The ratio of ends of team-tactical plays and uncontested shots were the independent variables included in the CHAID model (Figure 1), explaining that 69.5% of data were correctly classified. The model successfully classified 8944 of the 17,742 efficient ends of ball possession (50.4%) and successfully classified 17,899 of the 20,898 inefficient ends of the ball possession (85.6%). It was determined that the risk assessment of presumption accuracy of our CHAID model is (30.3%)—the amount of potentially misclassified evaluations (efficient, inefficient). It is very important to note that fouls received (Node 4), fast break (Node 11), cuts (Node 15, Node 16), P&R screener (Node 9), and transition and offensive rebound (Node 8) make up 30% of all nodes (*n* = 11592), and the response makes up for 75.4%. These four attributes (fouls received, fast break, cuts, and P&R screener) occur during 49.3% of all efficient ends of ball possession.

The predictive model enlisted four Parent Nodes—2 pts field goals, 3 pts field goals, fouls received, and turnovers. It makes sense from the perspective of the efficient end of possession that fouls received (Node 4) is the most efficient (response 100%), and 10.1% of all inspected cases are classified as this node. The second most efficient end of the ball possession is possessions in the 2 pts field goals (Node 1) terminal node fast break (Node 11), which has a response of 78.2%. Another efficient end of ball possession—cuts (Node 5). When analyzing terminal nodes, we can see that the probability of an efficient end of the ball possession increases if a cut happens in a shortest possible time. For cuts that happen in under 11 s, the level of response is 71.2% (Node 15). Response level of P&R screener (Node 9) is 61.5%, and the level of response of transition and offensive rebound (Node 8)—57.4%; however, the impact of time to these ends of the ball possession was not determined.

## 4. Discussion

### 4.1. Most Efficient Ends of the Ball Possession

The present study aimed to analyze the efficiency of the ends of the ball possession during team-tactical plays according to the outcomes of the end of the ball possession during team-tactical plays and possession duration of all the teams of an elite male Euroleague. Our findings indicated that ends of possession in the 2-point field goals were used more frequently—47.9% of the total, (successful 52.9%), than ends of possession in the 3-point field goals—28.2% of the total (successful 37.3%). In elite level competition, ends of possession during team-tactical plays have a big impact on the result of the game [7,13,34]. The present study addresses offense tactics during the endgame of close matches in men’s professional basketball [10]. Many researchers [13,35] look for answers that would help to win games and would allow the players to display the best of their abilities. A previous study determined which finishing actions were the most common during the European Championship (2013): screen on a player without the ball, handoff, pick and roll, put back, cut, spot up, and differential isolation situations [7]. Most successful finishing actions of Euroleague (2010–2011) players were: isolation, pick and roll, spot up, and cut [27].

Our research found that elite Euroleague teams usually end the ball possessions in 2-point field goals. The most efficient ends of the ball possession are fast break, transition, off rebound, cuts, and P&R screener situations. The higher efficiency of ends of the ball possession after a cut occurs until the 11th second of the possession. Our study confirms the fact that it is efficient to move without the ball: cut towards or away from the basket, change of direction, and different fake moves. On offense, the teamwork of two players when cuts is used has the highest efficiency (ES = 0.53 large). The player’s actions previously passing the ball were further successful if combined and synchronized with the receivers’ displacements, especially when cutting to the basket [7,10,36]. Basketball is a fast-paced and free-flowing sport, in which players’ actions and decisions continuously impact their teams’ prospective game outcomes [37].

The ends of the ball possessions are very effective after an offensive rebound [2]. A double advantage is gained by successfully grabbing an offensive rebound. Besides scoring points, they significantly reduce the opponent’s chance to create a quick and favorable transition. A regained possession of the ball logically increases a shot opportunity for the offense [15,16,38].

Our results showed that teams usually choose to end the ball possession in P&R in different ways. The researchers specify that the on-ball screen is the most frequently performed finishing action [19,35,39]. In modern basketball, P&R is an integral part of the game that is used at every level of the basketball competition [8,39,40]. Elite men basketball teams more often use P&R ball handler as an end of the ball possession, but its efficiency is way lower than of P&R screener situations [7]. Pass is the second most important technical action in the game after shooting [41,42], because the more accurate the pass, the better the opportunity that is created [2]. A previous study determined that ball-screen finishing actions such as pick and roll, pick and pop, and handoff make up 15% of all finishing actions. On the other hand, it was determined that 25.3% of total offenses finish with pick and roll [9].

We found that the players that choose Isolation as an end of the ball possession and attack either from the 2-point or the 3-point field goal area are usually inefficient. Finishing skills of the players are not suitable for important tactical plays, but the effectiveness of individual finishing actions determine the game more and more often [43]. Players that can efficiently play in isolation situations always tend to have a high role in the team’s tactical plays [44]. A previous study determined that 21.4% of Euroleague teams that were examined during the 2010–2011 playoffs and 25.3% of NBA (2010–2011) teams end all of their possessions by playing one on one [43]. Other studies analyzing the European Championship in Slovenia (2013) determined that 24% of all possessions end in Isolation. Playing 1 on 1 and spot-up is a great representation of the way basketball is played in the NBA. Particularly in the NBA, players have great 1 on 1 skills and are extremely athletic, with the optimal jump, speed, and power skills, making them dangerous when approaching the basket [45,46].

In our study, it was determined that the most frequently used finishing action of 3-point field goals in men’s Euroleague is a spot up (51%). The most shots were taken after a pass and that shows that the teams look to exploit the defensive mistakes using team-tactical plays. The most efficiently used end of possession from the 3-point field goal area was P&R ball handler (16%). The players with the ball efficiently exploit P&R situations and can attack from the range themselves.

The fouls received is an important tactical part of the game and we determined that during offense, 10.1% of all the ball possessions are stopped by a foul by the defender. P&R ball handler, post up, and spot-up situations were the ones the defending team stopped the most. Fouls are an effective way to recover ball possession [47]. European coaches tend to use foul tactics to interfere with the leading team’s game rhythm and fixed technical tactics during European basketball games [13].

The current results show that men’s teams mostly turn the ball over on offense (25.0%) when using P&R ball handler as an end other ball possession. Therefore, the analysts and the coaches have to perform deeper analysis and find out why their players turn the ball over so much in this possession [48]. Claims that the turnovers are created by bad offensive decisions or good defensive decision depend on the given situation.

### 4.2. The Benefit of Predictive Model

Our predictive model of efficient ends of the ball possession showed that the most effective ends of ball possessions in elite men’s basketball teams are fast break, cuts, P&R screener, transition, and offensive rebound. This study provides new information about the efficiency of the ball possessions of individual and team-tactical plays by playing in the 2-point field goal area. It also states that offensive efficiency depends on the balance between the outside game and the post-game [1,2,8]. However, the efficiency of ends of the ball possession depends on the player’s skills, as the better the players move and cooperate, the more likely the attack succeeds. Players’ dynamics are determined by specific offensive aims such as creating free space to pass and shoot, enhancing effective scoring options, and minimizing defensive pressure [6,10,19]. Cooperation between players, understanding each other, and good decision making are very important for the end of the ball possession to be efficient [10,49].

Moreover, results reported in this study showed that the duration of the ball possession is a significant factor to the efficiency of the entire possession. Our findings revealed that certain ends of the ball possession are most likely to be efficient if completed in a certain amount of time: cuts are recommended to be played (≤11 s, successful 71.2%), handoff (≤14 s, successful 55.1%), spot-up, P&R ball handler, off-screen (≤9 s, successful 52.3%), and isolation (≤21 s, successful 43.8%). Therefore, in elite men’s basketball, the most efficient ball possessions are those that last no more than 10 s. In the first half of the possession, the players are trying to decide on a tactical play to try to create space for open players (cuts, spot-up, fast break); thus, the use of screens is a group-tactical strategy used at the end of the ball possession [17,19].

## 5. Conclusions

This study showed the most common and the most efficient ends of the ball possession from the 2-point field goal area. Moreover, the most efficient types of the ends of the ball possession were showed: fast break, cuts, pick and roll screener, transition, and offensive rebound. A predictive model distinguished the most effective amount of time during which the end of the ball possession is executed: cuts (≤11 s), handoff (≤14 s), spot up, pick and roll ball handler, off-screen (≤9 s), and isolation (≤21 s).

The determined practical implications for coaches and researchers can be used to establish the demands of the game and to create training plans that can improve the player’s understanding of play types. Tactical training can be oriented towards the strict execution of technical elements, player decision making, coordinated offense results, and greater effectiveness of ends of the ball possession. Information of the study can be used by the analysts to predict and project play types regarding the duration of the possession and efficiency of ends of the ball possession.

## Figures and Tables

**Table 1:** Definition of outcomes and ends of the ball possession play types.

Variables	Description
Outcomes	
2-point field goals	Made or missed 2-point field goals
3-point field goals	Made or missed 3-point field goals
Fouls Received	Offensive plays ended with a foul drawn
Turnovers	A turnover is a mistake made by the offensive player or team that results in the defensive team gaining possession of the ball
End of the ball possession	
Handoff	The player hands out the ball to another player, which uses the passer’s screen to make a shot or to penetrate to the basket
Post up	Finishing action with the player’s back to the basket in the low post area
Spot up	Penetration or a shot after a pass to a player who is not strictly guarded or is open
Pick and Roll ball handler (P&R ball handler)	Screen set on the ball handler’s assigned defender
Pick and Roll screener (P&R screener)	Screener rolls to the rim or rolls away
Isolation	Finishing action with a shot or a penetration after play type 1 × 1
Cuts	Inside cut or outside cut and finishing action with a shot or penetration after a pass
Offensive rebound	An attacker recovers the ball after a missed-shot
Offscreen	The off-ball screen creates enough space for open shot or penetration
Transition	Beginning and finishing the attack within 5–8 s and creating a shot opportunity before opponent’s halfcourt defence is set
Fast break	Primary offense (1 × 0; 1 × 1; 2 × 1; 2 × 2; 3 × 2; 3 × 1; 3 × 3; 4 × 2) or secondary breaks (4 × 3; 4 × 4; 5 × 4) and those performed with an equal (attacking vs. defending team) or unequal (superiority for the attacking team) number of players, finishing within 5 s
Other actions	Quick-lost ball and other actions that cannot be classified into either of the above-mentioned finishing actions (offensive foul, half-court or longer shots, technical foul)

**Table 2:** Description of the ends of the ball possession during the game.

Variables	2-Point Field Goals							3-Point Field Goals							Fouls Received			Turnovers		
	Successful		Unsuccessful		95% CI	*p*-Value	ES	Successful		Unsuccessful		95% CI	*p*-Value	ES	Successful		95% CI	Unsuccessful		95% CI
	Mean	SD	Mean	SD				Mean	SD	Mean	SD				Mean	SD		Mean	SD	
Handoff	0.61	0.80	0.69	0.83	0.59–0.80	0.181	0.06	0.47	0.75	0.85	0.99	0.37–0.56	<0.001	0.21	0.33	0.59	0.25–0.40	0.50	0.79	0.40–0.60
Post up	4.20	2.24	4.60	2.27	4.31–4.88	0.080	0.08	0.03	0.22	0.04	0.25	0.003–0.05	0.363	0.04	1.88	1.52	1.68–2.07	2.37	1.63	2.16–2.57
Spot up	4.57	2.32	5.66	2.27	5.37–5.95	<0.001	0.23	9.18	0.22	14.18	4.32	8.73–9.63	<0.001	0.55	1.70	1.52	1.50–1.89	2.57	1.71	2.35–2.79
P&R ball handler	6.36	2.89	7.54	3.35	7.11–7.96	<0.001	0.18	2.60	1.98	4.55	2.30	2.35–2.86	<0.001	0.43	2.48	1.53	2.29–2.68	5.60	2.42	5.29–5.90
P&R screener	4.57	2.24	2.86	1.84	2.62–3.09	<0.001	0.39	0.53	0.77	0.88	1.04	0.44–0.63	<0.001	0.18	1.27	1.25	1.11–1.43	0.89	0.95	0.77–1.01
Isolation	2.41	1.66	3.45	2.14	3.17–3.72	<0.001	0.25	0.83	0.89	2.16	1.44	0.72–0.94	<0.001	0.49	1.10	1.17	0.96–1.25	1.00	1.11	0.86–1.15
Cuts	6.58	2.82	3.58	2.01	3.32–3.84	<0.001	0.53	0.01	0.13	0.03	0.24	−0.01–0.02	0.316	0.05	1.43	1.24	1.28–1.59	0.79	0.94	0.67–0.91
Offensive rebound	4.01	2.20	2.99	1.82	2.76–3.22	<0.001	0.23	0.01	0.11	0.03	0.22	−0.002–0.03	0.7	0.02	1.00	1.04	0.86–1.13	0.60	0.81	0.50–0.71
Off screen	1.43	1.28	1.79	1.37	1.62–1.97	0.003	0.14	1.78	1.54	2.82	1.88	1.59–1.98	<0.001	0.29	0.40	0.67	0.32–0.49	0.73	0.89	0.61–0.84
Transition	3.10	1.77	2.29	1.67	2.08–2.50	<0.001	0.24	1.43	1.20	2.28	1.62	1.28–1.59	<0.001	0.27	1.51	1.21	1.35–1.66	1.98	1.39	1.80–2.16
Fast break	2.79	1.86	0.78	0.90	0.66–0.89	<0.001	0.60	0.05	0.21	0.05	0.06	0.02–0.07	0.84	0.01	0.48	0.68	0.40–0.57	0.08	0.40	0.03–0.13
Other actions	0.15	0.58	0.14	0.38	0.09–0.19	0.425	0.04	0.02	0.14	0.64	0.78	0.03–0.4	<0.001	0.53	2.63	1.86	2.40–2.87	5.12	2.44	4.81–5.43

Note: SD: standard deviations; CI: confidence interval; ES: effect size.

## Data Availability

Not applicable.
